# Malignant Hyperthermia and Idiopathic HyperCKemia

**DOI:** 10.1155/2011/194296

**Published:** 2011-11-23

**Authors:** Pashtoon Murtaza Kasi

**Affiliations:** International Scholars Program, Department of Internal Medicine, University of Pittsburgh Medical Center (UPMC), Pittsburgh, PA 15213, USA

## Abstract

Malignant hyperthermia (MH) is a rare but life-threatening condition that is more frequently encountered and discussed within the anesthesia literature. Here we through a case specifically discuss the susceptibility of individuals and/or families with asymptomatic unexplained elevations of creatine kinase (CK), also frequently referred to as hyperCKemia or idiopathic hyperCKemia (IHCK) in recent reports. The clinical implications would be to underscore the importance of this as a susceptibility to developing MH and highlight the importance of genetic susceptibility testing in such cases. Anesthesiologists and critical care intensivists as well as primary care physicians should keep this in mind when seeing patients with asymptomatic hyperCKemia and potentially inform them about the possibility of developing MH if exposed to triggering agents. Genetic susceptibility testing should be considered if available and family members should also receive nontriggering agents when undergoing anesthesia and wear Medic Alert tags.

## 1. Introduction

Malignant hyperthermia (MH) is a rare but life-threatening condition that is more frequently encountered and discussed within the anesthesia literature [1]. Here we through a case specifically discuss the susceptibility of individuals and/or families with asymptomatic unexplained elevations of creatine kinase (CK) or creatine phosphokinase (CPK), also frequently referred to as HyperCKemia or idiopathic hyperCKemia (IHCK) in recent reports [2]. The clinical implications would be underscoring the importance of this as a susceptibility to developing MH. Anesthesiologists and critical care intensivists as well as primary care physicians should keep this in mind when seeing patients with asymptomatic hyperCKemia and potentially inform them about the possibility of developing MH if exposed to triggering agents.

## 2. Case Presentation

An 18-year-old man with past medical history significant only for asymptomatic creatine phosphokinase (CPK) elevation (familial) was transferred to our medical intensive care unit after developing malignant hyperthermia (MH) intraoperatively at an outside hospital. 

History of his presenting illness revealed that the patient was in his usual state of health till a day prior to admission when he got injured at a local high school football game. He was noted to have a right tibia-fibula fracture and underwent IM nailing (rodding) of the right tibia by orthopedics. 

Review of his operative records revealed that early in the course of his surgery, it was noted that patient's end tidal carbon dioxide concentration (ETCO2) was noted to have increased from 42 to 100 mm Hg during the course of the surgery and his temperature went up to >104 (apparently was not measureable) following which concern was raised for malignant hyperthermia (MH). 

Labs were consistent for acidosis (pH 7.29) and hyperkalemia (K^+^-7 mMol/L). Patient was given dantrolene IV (recommended initial dose being 2.5 mg/kg) immediately along with calcium gluconate, kayexalate, insulin/dextrose 50% and albuterol as well for the hyperkalemia and transferred to our facility for further management and care. Cooling blankets, fluids, and acetaminophen were also concomitantly administered to bring his temperature down.

Dantrolene (1 mg/kg) IV was continued till hyperthermia resolved and was then transitioned to dantrolene PO 6 mg/kg in 4 divided doses for the next 3 days. CPK levels peaked up to 3922 IU/L initially when the patient had presented and subsequently decreased during the course of his stay. 

In terms of medications charted at the outside hospital, patient got sevoflurane, propofol, lidocaine, labetalol, rocuronium, fentanyl, and versed. It was presumed that this would have been likely secondary to sevoflurane (one of the volatile gaseous inhalation anesthetics which is a known trigger to MH in susceptible individuals) [4–6]. 

Once we were able to talk to the family, it revealed a very interesting family history. Apparently patient's father was noted to have CPK elevations after being started on a statin, but these levels remained elevated despite the discontinuation of the medication. It was at that point in time that the patient's primary care physician was concerned for a possible familial elevation and screened his family too, which also revealed high CPK levels. The highest CPK level of the patient's father has been up to 1600 IU/L. Patient's CPK level has been in the range of 500–600 (IU/L) and sisters' levels have been up to 1100 IU/L. No family history of malignant hyperthermia in the family or any history of any adverse reactions to medications was reported. Patient's family was extensively worked up for known genetic mutations to explain the high CPK levels along with screening for dystrophies and myopathies, but the results were inconclusive. 

The significance of this in the context of susceptibility to MH is discussed as follows.

## 3. HyperCKemia and Susceptibility to Malignant Hyperthermia

High creatine phosphokinase (CPK) levels usually signify underlying myopathies or muscle injury [3]. However, in some cases the cause is not identified and these patients continue to have asymptomatic elevations of the enzyme. This is referred to as idiopathic hyperCKemia (IHCK) and classically is defined by “at least 3 serum CK levels more than twice normal that remain increased over at least 3 months in patients with no evidence of neuromuscular disease” [2]. The condition is noted more often in males and appears to be familial (see Table 1). A high percentage of autosomal dominant cases have also been reported [7]. 

Reviewing the literature, it is interesting to note that these patients have been shown to be susceptible to developing malignant hyperthermia. Usually this is determined by *in vitro* contracture testing, which is considered the gold standard for assessing malignant hyperthermia susceptibility (MHS) [8]. 

Table 1 outlines the outcomes of 2 studies specifically assessing the susceptibility to malignant hyperthermia (MHS) in IHCK. As evident, the incidence is noted to vary widely and may also depend on the selection of cases selected for testing. However, it is important to note that the test is not 100% sensitive in ruling out susceptibility to developing MH; thus, the true incidence of developing MH in patients with idiopathic hyperCKemia is not known [3]. 

At the same time it is argued by some reports that it is questionable if patients with asymptomatic elevations of CPK should undergo MH susceptibility testing especially if they do not have a family history of anesthesia-related complications, given the incidence is variable and sometimes can also lead to false positives [3]. However, in our opinion with the fact that the condition can be life threatening, and as seen in our case as well, patient not having a family history of any anesthesia-related complications, we would recommend susceptibility testing if available. 

We sent our patient's blood work for testing at UPMC laboratories and the sequence analysis identified a heterozygous nucleotide substitution of C to T at nucleotide 487 [c.487C > T] in exon 6 of the RYR1 gene (type 1 ryanodine receptor gene, a gene for calcium channel of skeletal muscle which is located on chromosome 19q13.1). The change involves the 1st nucleotide of codon 163 and results in a missense amino acid variant: [p.Arg163Cys] that is known to cause the disease. Per laboratory data here, it is estimated that more than 50% of the malignant hyperthermia cases are located in regions where many of the reported mutations are clustered. 

Furthermore, in this day and age with numerous other alternatives available from anesthesia standpoint, it would not be reasonable to take the risk of exposing patients to triggering agents even if the risk is small.  Table 2 summarizes some of the known triggering agents for MH along with classes of drugs that have been questionably linked to MH. 

As advocated by some authors, we would also recommend that family members receive nontriggering agents when undergoing anesthesia and wear Medic Alert tags [2, 9].

The establishment of registries to keep track of such rare disorders and conduct research would be helpful in not only providing diagnostic testing but also in further elucidating the pathogenetic mechanisms responsible for the life-threatening complications seen [10]. 

## Figures and Tables

**Table 1:** Summary of 2 studies specifically studying the susceptibility of developing MH in patients with unexplained hyperCKemia.

Study	Sample size	Testing	Outcome
(1) Malandrini et al. [3]	37 (i) 51.1% were asymptomatic (ii) 26 males and 11 females	*In vitro* contracture testing with halothane-caffeine	1 MH susceptible (2.7%) and 1 MH equivocal patient No familial cases noted in this study

(2) Weglinski et al. [2]	49 (i) All were asymptomatic (ii) 36 males and 13 females	*In vitro* contracture testing with halothane-caffeine	24 (49%) had positive contracture tests 14 patients had a family history of high CK levels

**Table 2:** Groups of drugs noted to cause or associated with malignant hyperthermia [10, 11].

Inhaled general anesthetics	Depolarizing muscle relaxants	Other classes of drugs that show inconclusive evidence/case reports [12]
Sevoflurane**	Succinylcholine	Serotonergic drugs
Desflurane		Statins
Enflurane		Ondansetron
Chloroform		Methylene blue
(Trichloromethane, methyl trichloride)		Phosphodiesterase III inhibitors
Cyclopropane		Tetracaine
Ether Halothane Isoflurane Methoxyflurane Trichloroethylene Xenon		

**Trigger in our patient.
